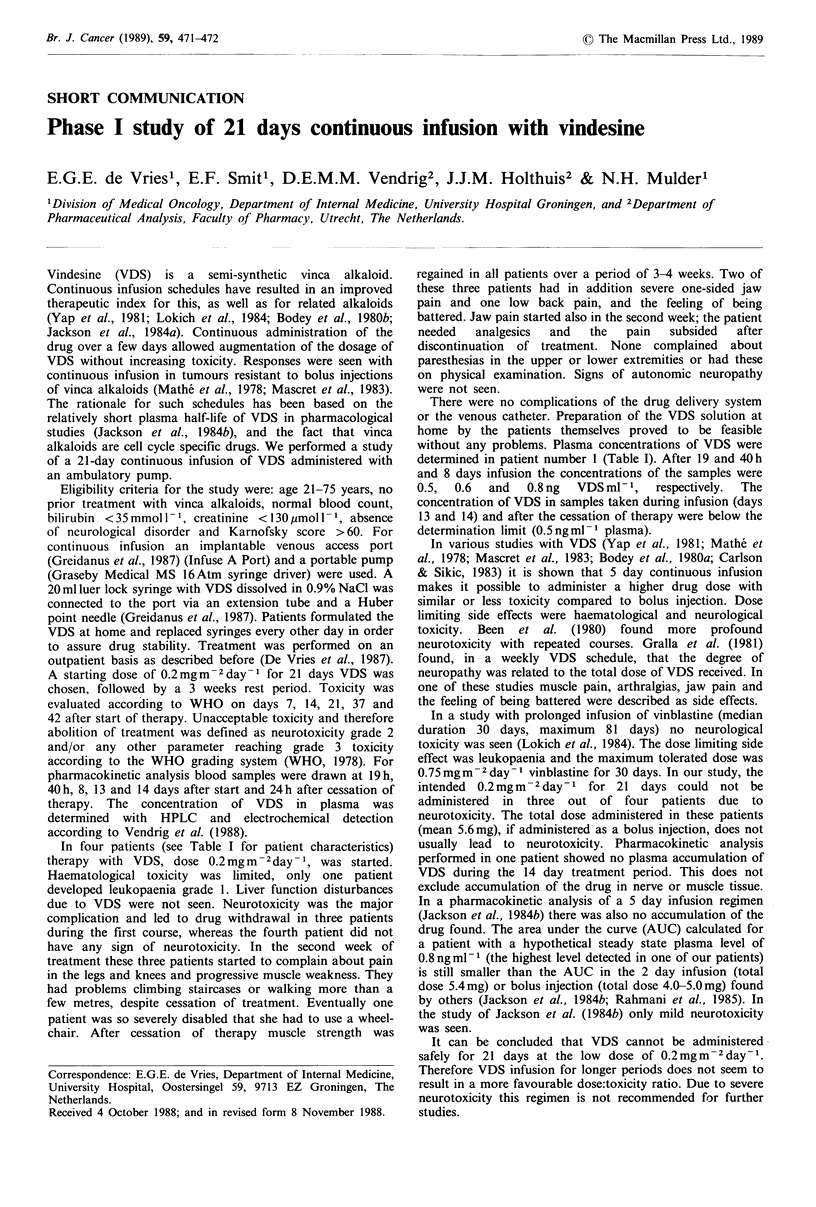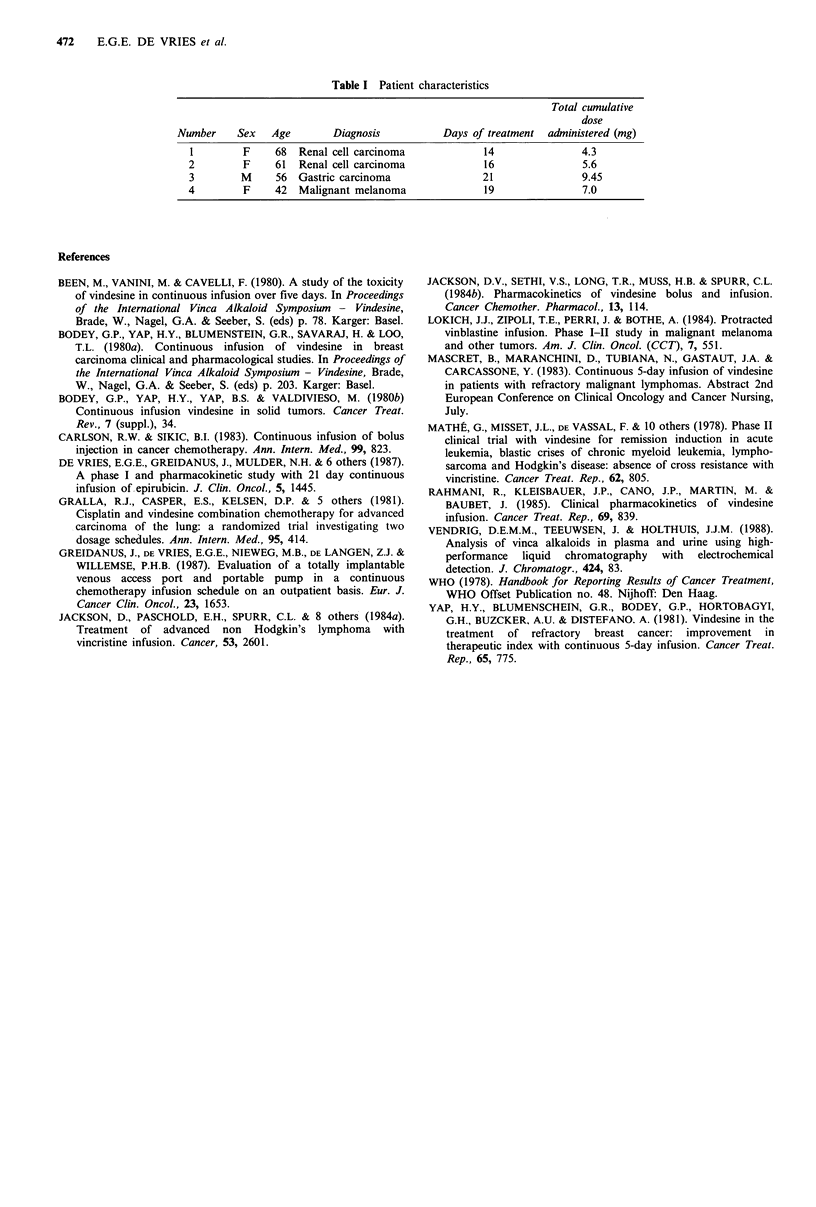# Phase I study of 21 days continuous infusion with vindesine.

**DOI:** 10.1038/bjc.1989.97

**Published:** 1989-03

**Authors:** E. G. de Vries, E. F. Smit, D. E. Vendrig, J. J. Holthuis, N. H. Mulder

**Affiliations:** Department of Internal Medicine, University Hospital Groningen, The Netherlands.


Br. J. Cancer (1989), 59, 471–472 © The Macmillan Press Ltd., 1989

SHORT COMMUNICATION

### Phase I study of 21 days continuous infusion with vindesine

E.G.E. de Vries[1], E.F. Smit[1], D.E.M.M. Vendrig[2], J.J.M. Holthuis[2] & N.H. Mulder[1]

[1]*Division of Medical Oncology, Department of Internal Medicine, University Hospital Groningen, and* [2]*Department of Pharmaceutical Analysis, Faculty of Pharmacy, Utrecht, The Netherlands.*

Vindesine (VDS) is a semi-synthetic vinca alkaloid. Continuous infusion schedules have resulted in an improved therapeutic index for this, as well as for related alkaloids (Yap *et al.*, 1981; Lokich *et al.*, 1984; Bodey *et al.*, 1980*b*; Jackson *et al.*, 1984*a*). Continuous administration of the drug over a few days allowed augmentation of the dosage of VDS without increasing toxicity. Responses were seen with continuous infusion in tumours resistant to bolus injections of vinca alkaloids (Mathé *et al.*, 1978; Mascret *et al.*, 1983). The rationale for such schedules has been based on the relatively short plasma half-life of VDS in pharmacological studies (Jackson *et al.*, 1984*b*), and the fact that vinca alkaloids are cell cycle specific drugs. We performed a study of a 21-day continuous infusion of VDS administered with an ambulatory pump.

Eligibility criteria for the study were: age 21–75 years, no prior treatment with vinca alkaloids, normal blood count, bilirubin $<35\,\mathrm{mmol\,l^{-1}}$, creatinine $<130\,\mu\mathrm{mol\,l^{-1}}$, absence of neurological disorder and Karnofsky score >60. For continuous infusion an implantable venous access port (Greidanus *et al.*, 1987) (Infuse A Port) and a portable pump (Graseby Medical MS 16Atm syringe driver) were used. A 20 ml luer lock syringe with VDS dissolved in 0.9% NaCl was connected to the port via an extension tube and a Huber point needle (Greidanus *et al.*, 1987). Patients formulated the VDS at home and replaced syringes every other day in order to assure drug stability. Treatment was performed on an outpatient basis as described before (De Vries *et al.*, 1987). A starting dose of $0.2\,\mathrm{mg\,m^{-2}\,day^{-1}}$ for 21 days VDS was chosen, followed by a 3 weeks rest period. Toxicity was evaluated according to WHO on days 7, 14, 21, 37 and 42 after start of therapy. Unacceptable toxicity and therefore abolition of treatment was defined as neurotoxicity grade 2 and/or any other parameter reaching grade 3 toxicity according to the WHO grading system (WHO, 1978). For pharmacokinetic analysis blood samples were drawn at 19 h, 40 h, 8, 13 and 14 days after start and 24 h after cessation of therapy. The concentration of VDS in plasma was determined with HPLC and electrochemical detection according to Vendrig *et al.* (1988).

In four patients (see Table I for patient characteristics) therapy with VDS, dose $0.2\,\mathrm{mg\,m^{-2}\,day^{-1}}$, was started. Haematological toxicity was limited, only one patient developed leukopaenia grade 1. Liver function disturbances due to VDS were not seen. Neurotoxicity was the major complication and led to drug withdrawal in three patients during the first course, whereas the fourth patient did not have any sign of neurotoxicity. In the second week of treatment these three patients started to complain about pain in the legs and knees and progressive muscle weakness. They had problems climbing staircases or walking more than a few metres, despite cessation of treatment. Eventually one patient was so severely disabled that she had to use a wheelchair. After cessation of therapy muscle strength was regained in all patients over a period of 3–4 weeks. Two of these three patients had in addition severe one-sided jaw pain and one low back pain, and the feeling of being battered. Jaw pain started also in the second week; the patient needed analgesics and the pain subsided after discontinuation of treatment. None complained about paresthesias in the upper or lower extremities or had these on physical examination. Signs of autonomic neuropathy were not seen.

There were no complications of the drug delivery system or the venous catheter. Preparation of the VDS solution at home by the patients themselves proved to be feasible without any problems. Plasma concentrations of VDS were determined in patient number 1 (Table I). After 19 and 40 h and 8 days infusion the concentrations of the samples were 0.5, 0.6 and $0.8\,\mathrm{ng\ VDS\,ml^{-1}}$, respectively. The concentration of VDS in samples taken during infusion (days 13 and 14) and after the cessation of therapy were below the determination limit ($0.5\,\mathrm{ng\,ml^{-1}}$ plasma).

In various studies with VDS (Yap *et al.*, 1981; Mathé *et al.*, 1978; Mascret *et al.*, 1983; Bodey *et al.*, 1980*a*; Carlson & Sikic, 1983) it is shown that 5 day continuous infusion makes it possible to administer a higher drug dose with similar or less toxicity compared to bolus injection. Dose limiting side effects were haematological and neurological toxicity. Been *et al.* (1980) found more profound neurotoxicity with repeated courses. Gralla *et al.* (1981) found, in a weekly VDS schedule, that the degree of neuropathy was related to the total dose of VDS received. In one of these studies muscle pain, arthralgias, jaw pain and the feeling of being battered were described as side effects.

In a study with prolonged infusion of vinblastine (median duration 30 days, maximum 81 days) no neurological toxicity was seen (Lokich *et al.*, 1984). The dose limiting side effect was leukopaenia and the maximum tolerated dose was $0.75\,\mathrm{mg\,m^{-2}\,day^{-1}}$ vinblastine for 30 days. In our study, the intended $0.2\,\mathrm{mg\,m^{-2}\,day^{-1}}$ for 21 days could not be administered in three out of four patients due to neurotoxicity. The total dose administered in these patients (mean 5.6 mg), if administered as a bolus injection, does not usually lead to neurotoxicity. Pharmacokinetic analysis performed in one patient showed no plasma accumulation of VDS during the 14 day treatment period. This does not exclude accumulation of the drug in nerve or muscle tissue. In a pharmacokinetic analysis of a 5 day infusion regimen (Jackson *et al.*, 1984*b*) there was also no accumulation of the drug found. The area under the curve (AUC) calculated for a patient with a hypothetical steady state plasma level of $0.8\,\mathrm{ng\,ml^{-1}}$ (the highest level detected in one of our patients) is still smaller than the AUC in the 2 day infusion (total dose 5.4 mg) or bolus injection (total dose 4.0–5.0 mg) found by others (Jackson *et al.*, 1984*b*; Rahmani *et al.*, 1985). In the study of Jackson *et al.* (1984*b*) only mild neurotoxicity was seen.

It can be concluded that VDS cannot be administered safely for 21 days at the low dose of $0.2\,\mathrm{mg\,m^{-2}\,day^{-1}}$. Therefore VDS infusion for longer periods does not seem to result in a more favourable dose:toxicity ratio. Due to severe neurotoxicity this regimen is not recommended for further studies.

Correspondence: E.G.E. de Vries, Department of Internal Medicine, University Hospital, Oostersingel 59, 9713 EZ Groningen, The Netherlands.
Received 4 October 1988; and in revised form 8 November 1988.

**Table I** Patient characteristics

| *Number* | *Sex* | *Age* | *Diagnosis* | *Days of treatment* | *Total cumulative dose administered (mg)* |
|---|---|---|---|---|---|
| 1 | F | 68 | Renal cell carcinoma | 14 | 4.3 |
| 2 | F | 61 | Renal cell carcinoma | 16 | 5.6 |
| 3 | M | 56 | Gastric carcinoma | 21 | 9.45 |
| 4 | F | 42 | Malignant melanoma | 19 | 7.0 |

### References

BEEN, M., VANINI, M. & CAVELLI, F. (1980). A study of the toxicity of vindesine in continuous infusion over five days. In *Proceedings of the International Vinca Alkaloid Symposium – Vindesine*, Brade, W., Nagel, G.A. & Seeber, S. (eds) p. 78. Karger: Basel.

BODEY, G.P., YAP, H.Y., BLUMENSTEIN, G.R., SAVARAJ, H. & LOO, T.L. (1980*a*). Continuous infusion of vindesine in breast carcinoma clinical and pharmacological studies. In *Proceedings of the International Vinca Alkaloid Symposium – Vindesine*, Brade, W., Nagel, G.A. & Seeber, S. (eds) p. 203. Karger: Basel.

BODEY, G.P., YAP, H.Y., YAP, B.S. & VALDIVIESO, M. (1980*b*) Continuous infusion vindesine in solid tumors. *Cancer Treat. Rev.*, 7 (suppl.), 34.

CARLSON, R.W. & SIKIC, B.I. (1983). Continuous infusion of bolus injection in cancer chemotherapy. *Ann. Intern. Med.*, **99**, 823.

DE VRIES, E.G.E., GREIDANUS, J., MULDER, N.H. & 6 others (1987). A phase I and pharmacokinetic study with 21 day continuous infusion of epirubicin. *J. Clin. Oncol.*, **5**, 1445.

GRALLA, R.J., CASPER, E.S., KELSEN, D.P. & 5 others (1981). Cisplatin and vindesine combination chemotherapy for advanced carcinoma of the lung: a randomized trial investigating two dosage schedules. *Ann. Intern. Med.*, **95**, 414.

GREIDANUS, J., DE VRIES, E.G.E., NIEWEG, M.B., DE LANGEN, Z.J. & WILLEMSE, P.H.B. (1987). Evaluation of a totally implantable venous access port and portable pump in a continuous chemotherapy infusion schedule on an outpatient basis. *Eur. J. Cancer Clin. Oncol.*, **23**, 1653.

JACKSON, D., PASCHOLD, E.H., SPURR, C.L. & 8 others (1984*a*). Treatment of advanced non Hodgkin's lymphoma with vincristine infusion. *Cancer*, **53**, 2601.

JACKSON, D.V., SETHI, V.S., LONG, T.R., MUSS, H.B. & SPURR, C.L. (1984*b*). Pharmacokinetics of vindesine bolus and infusion. *Cancer Chemother. Pharmacol.*, **13**, 114.

LOKICH, J.J., ZIPOLI, T.E., PERRI, J. & BOTHE, A. (1984). Protracted vinblastine infusion. Phase I–II study in malignant melanoma and other tumors. *Am. J. Clin. Oncol. (CCT)*, **7**, 551.

MASCRET, B., MARANCHINI, D., TUBIANA, N., GASTAUT, J.A. & CARCASSONE, Y. (1983). Continuous 5-day infusion of vindesine in patients with refractory malignant lymphomas. Abstract 2nd European Conference on Clinical Oncology and Cancer Nursing, July.

MATHÉ, G., MISSET, J.L., DE VASSAL, F. & 10 others (1978). Phase II clinical trial with vindesine for remission induction in acute leukemia, blastic crises of chronic myeloid leukemia, lymphosarcoma and Hodgkin's disease: absence of cross resistance with vincristine. *Cancer Treat. Rep.*, **62**, 805.

RAHMANI, R., KLEISBAUER, J.P., CANO, J.P., MARTIN, M. & BAUBET, J. (1985). Clinical pharmacokinetics of vindesine infusion. *Cancer Treat. Rep.*, **69**, 839.

VENDRIG, D.E.M.M., TEEUWSEN, J. & HOLTHUIS, J.J.M. (1988). Analysis of vinca alkaloids in plasma and urine using high-performance liquid chromatography with electrochemical detection. *J. Chromatogr.*, **424**, 83.

WHO (1978). *Handbook for Reporting Results of Cancer Treatment*, WHO Offset Publication no. 48. Nijhoff: Den Haag.

YAP, H.Y., BLUMENSCHEIN, G.R., BODEY, G.P., HORTOBAGYI, G.H., BUZCKER, A.U. & DISTEFANO, A. (1981). Vindesine in the treatment of refractory breast cancer: improvement in therapeutic index with continuous 5-day infusion. *Cancer Treat. Rep.*, **65**, 775.